# Connexin 50 Regulates Surface Ball-and-Socket Structures and Fiber Cell Organization

**DOI:** 10.1167/iovs.16-19521

**Published:** 2016-06-09

**Authors:** Eddie Wang, Andrew Geng, Ankur M. Maniar, Byron W. H. Mui, Xiaohua Gong

**Affiliations:** School of Optometry and Vision Science Program University of California Berkeley, Berkeley, California, United States

**Keywords:** connexin 50, connexin 46, gap junction, cataract, lens fiber cell

## Abstract

**Purpose:**

The roles of gap junction protein connexin 50 (Cx50) encoded by *Gja8*, during lens development are not fully understood. Connexin 50 knockout (KO) lenses have decreased proliferation of epithelial cells and altered fiber cell denucleation. We further investigated the mechanism for cellular defects in Cx50 KO (*Gja8^−/−^)* lenses.

**Methods:**

Fiber cell morphology and subcellular distribution of various lens membrane/cytoskeleton proteins from wild-type and Cx50 KO mice were visualized by immunofluorescent staining and confocal microscopy.

**Results:**

We observed multiple morphological defects in the cortical fibers of Cx50 KO lenses, including abnormal fiber cell packing geometry, decreased F-actin enrichment at tricellular vertices, and disrupted ball-and-socket (BS) structures on the long sides of hexagonal fibers. Moreover, only small gap junction plaques consisting of Cx46 (α3 connexin) were detected in cortical fibers and the distributions of the BS-associated beta-dystroglycan and ZO-1 proteins were altered.

**Conclusions:**

Connexin 50 gap junctions are important for BS structure maturation and cortical fiber cell organization. Connexin 50–based gap junction plaques likely form structural domains with an array of membrane/cytoskeletal proteins to stabilize BS. Loss of Cx50-mediated coupling, BS disruption, and altered F-actin in Cx50 KO fibers, thereby contribute to the small lens and mild cataract phenotypes.

The avascular lens consists of an epithelial monolayer at its anterior hemisphere and a bulk mass of concentrically layered fiber cells. New fibers differentiate from equatorial surface epithelial cells; they elongate with approximately hexagonal cross section and stack radially onto older fibers.^1–3^ As they mature, fibers fill with crystallin proteins and degrade their intracellular organelles to achieve a high refractive index with minimal light scattering.^4^ During lens fiber differentiation and maturation, extensive intercellular metabolic coupling mediated by gap junctions^5^ and physical coupling via surface interlocking membrane domains occurs.^6^ These interactions are critical to establishing and maintaining a lens' optical properties, including transparency and shape.

Intercellular gap junction channels provide pathways for metabolites, small signaling molecules, ions, and water between fibers and between fibers and anterior epithelial cells. The channels form by the docking of two hemichannels, which are hexameric oligomers of connexin subunits.^7^ Connexin 46 (Cx46) (α3 connexin) and connexin 50 (Cx50) (α8 connexin), encoded by the *Gja3* and *Gja8* genes, respectively, are the major subunits for gap junctions in mouse lens fibers.^5^ Connexins are essential for lens development and homeostasis as demonstrated by the various adverse effects, such as cataracts and growth defects, that occur in animals and humans due to Cx46/Cx50 knockouts (KO) and mutations.^8–13^ In particular, only Cx50 is critical for lens size. Connexin 50 KO (*Gja8^−/−^*) lenses are approximately 60% of the size of their wild-type (WT) counterparts. The lenses have a reduced population of proliferating epithelial cells, altered fiber cell denucleation, and mild nuclear cataracts.^9,10,14^ Knocking *Gja3* into the *Gja8* locus (Cx50KI46) prevented the cataract phenotype but only partly restored lens size.^15^ Subsequent studies showed that Cx50 indirectly interacts with growth-related signaling pathways, including MAPK and PI3K.^16,17^ Connexin 50 was also shown to form functional hemichannels^18^ and to promote lens fiber differentiation in vitro, independent of its channel-forming abilities.^19–21^ Currently, the molecular and cellular bases for the Cx50 KO lens phenotypes are not fully elucidated, nor is it fully understood how the Cx50 activities discovered in vitro function in the in vivo setting.

Peripheral lens fibers are physically coupled through specialized membrane structures known as ball-and-socket (BS) structures. Each BS consists of a portion of one cell membrane bulging into a neighboring invaginated membrane. They are mostly found connecting the long hexagonal faces between cortical lens fibers.^6^ Ball-and-sockets contain gap junction plaques that are structurally distinct from non–BS-associated plaques and are believed to provide rapid exchange of nutrients, ions, and water for fiber cell differentiation and elongation during lens growth.^22,23^ Currently, the mechanisms of BS maturation are unknown. Ball-and-sockets are distinct from protrusions, another class of membrane interlocking domains. Protrusions emanate from the vertices of hexagonal fiber cells, lack gap junctions, and are enriched with actin and aquaporin-0.^6,23,24^

In this study, we identified fiber cell morphogenesis defects, especially disruption of BS, in Cx50 KO cortical fibers. Our findings suggest that Cx50 gap junctions serve a structural role in maintaining BS structures as well as a role in lens fiber organization.

## Materials and Methods

### Animals

Mouse care and breeding were performed according to the Animal Care and Use Committee–approved animal protocol (University of California Berkeley, Berkeley, CA, USA) and the ARVO Statement for the Use of Animals in Ophthalmic and Vision Research. Wild-type and Cx50 KO (*Gja8^−/−^*) mice in a *CP49^+/+^* mixed C57BL/6J-129SvJae background were used. Connexin 46 KO (*Gja3^−/−^*) in both *CP49^+/+^* C57BL/6J and *CP49del/del* 129SvJae backgrounds were used.^25^

### Immunohistochemistry

Mouse eyes were immediately enucleated from euthanized animals, lenses were dissected in PBS and then immersed into 4% paraformaldehyde (PFA) in PBS at 37°C for 30 minutes. The fixed lenses were washed three times with PBS then sectioned into 150-μm slices with a vibratome (Leica VT1000 S; Leica, Wetlar, Germany). The slices were postfixed for 2 minutes in 4% PFA in PBS then washed three times with PBS before immunohistochemical analysis. A rabbit antibody against the intracellular loop of Cx46,^26^ a rabbit antibody against the C-terminal region of Cx50,^27^ a rabbit anti-βDystroglycan (anti-βDys) antibody (MANDAG2[7D11]; Developmental Studies Hybridoma Bank, Iowa City, IA, USA), and a rabbit anti-ZO-1 antibody (40-2200; Thermo-Fisher, Waltham, MA, USA) were used. Antigen retrieval was performed on sections destined for antibody labeling with the exception of anti-Cx50 labeling. The sections were placed in citrate buffer (10 mM sodium citrate, 0.05% vol/vol Tween 20) at 80°C for 30 minutes then washed three times with PBS. The sections were then permeabilized and blocked in PB solution (3% wt/vol BSA, 3% vol/vol normal goat serum, 0.3% vol/vol Triton X-100 in PBS) for 1 hour. Primary antibodies diluted 1:100 in PBS were applied overnight at 4°C. The sections were washed four times in 0.05% vol/vol Tween 20 in PBS then incubated overnight at 4°C or for 3 hours at 37°C with fluorescently labeled secondary antibodies (1:100 in PBS). Rhodamine-wheat germ agglutinin (WGA) (Vector Laboratories, Burlingame, CA, USA) (1:100) was added with secondary antibodies. Lens sections that did not undergo antigen retrieval were colabeled with rhodamine-WGA (1:100) and FITC-phalloidin (1:100) (Thermo-Fisher), and then washed with PBS four times before being mounted with VECTASHIELD Antifade Mounting Medium with 4′,6-diamidino-2-phenylindole (DAPI) (Vector Laboratories). Four or more samples were used to collect phalloidin staining data. Connexin 46, ZO-1, and βDys staining was performed on three or more independent samples with and without antigen retrieval. Representative images from antigen-retrieved samples are shown in the figures. Multiple images per sample were collected by confocal microscopy (LSM 700; Zeiss, Jena, Germany).

## Results

### Disrupted Fiber Cell Organization, Morphology, and F-Actin Distribution

**Figure 1:**
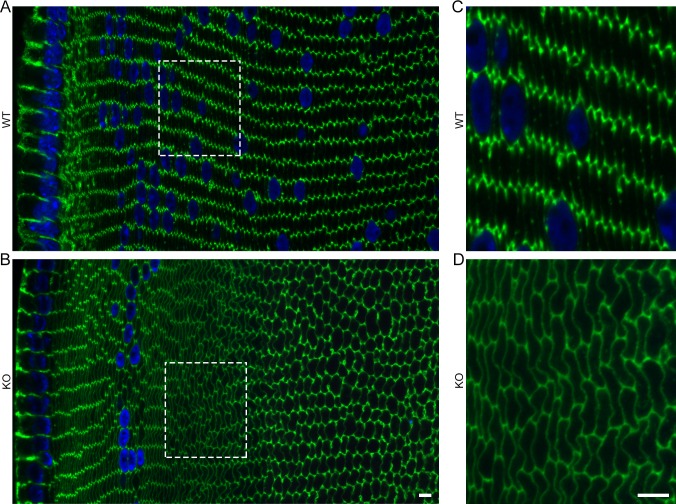
Fiber organization and F-actin distribution in WT and Cx50 KO lens fibers. Composite images of F-actin (*green*) and DAPI (*blue*)-stained cortical cross sections. The WT lens has regularly arranged, parallel columns of hexagonal fiber cells with enriched F-actin at cell vertices (**A**) and enlarged region in (**C**). Connexin 50 KO lens fibers are ordered near the surface but have areas with poorer organization approximately 80 μm from the periphery (**B**) and enlarged region in (**D**). Staining with F-actin also becomes more uniform around the cell boundaries. *Scale bars*: 5 μm.

We first characterized the morphology of Cx50 KO lens fibers. To do so, we examined cortical cross sections from 3-week-old WT and Cx50 KO mouse lenses by labeling F-actin with phalloidin (Fig. 1, Supplementary Fig. S1). Wild-type lens fibers were hexagonal in cross section and well organized into radially stacked columns with each cell being similar in dimensions to its neighbors (Figs. 1A, 1C). Connexin 50 KO lens fibers tended to be well organized near the surface, but were conspicuously more variable in size and shape in deeper cortical fibers (∼80 μm deep), leading to regions of irregularly packed cells (Figs. 1B, 1D). Wild-type fiber F-actin staining was considerably enriched at tricellular vertices (Figs. 1A, 1C), whereas KO F-actin staining was more uniform along the cell periphery, especially after approximately 50 μm in from the lens capsule (Figs. 1B, 1D).

### Disruption of Cx46 Gap Junction Plaques and Ball-and-Socket Structures

**Figure 2:**
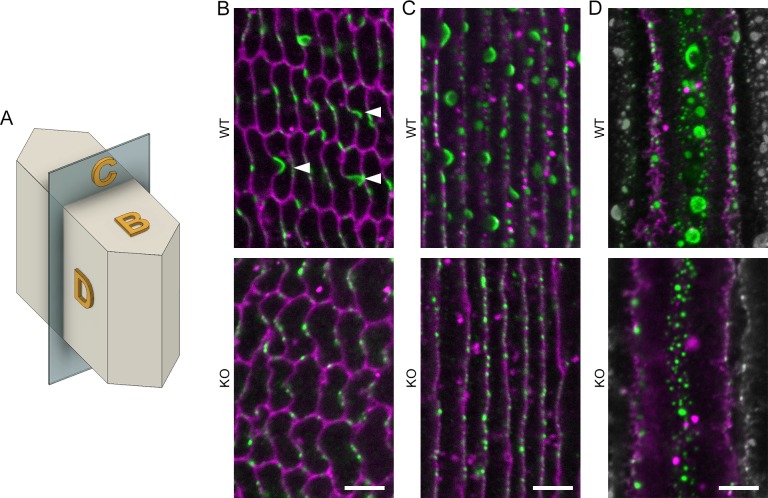
The distribution of Cx46 in WT and Cx50 KO lenses. (**A**) Drawing of a hexagonal-shaped fiber cell to clarify the orientation of the imaging planes in (**B**) to (**D**). (**B**) Cross section of WT fibers (*top*) shows Cx46 (*green*) staining is enriched on BS and corresponds to weak areas of WGA (*magenta*) staining (*arrowheads*). Cross section of Cx50 KO fibers (*bottom*) shows punctate staining on the membrane, but no enriched areas corresponding to BS. Images of a plane going through the broad sides of WT and Cx50 KO fibers (**C**) and onto the broad side surface of WT and Cx50 KO fibers (**D**) confirm lack of BS enriched with Cx46 staining in Cx50 KO fibers compared with WT fibers. Adjacent cells in (**D**) were changed to grayscale for clarity. (**B**) and (**C**) collected approximately 75 to 125 μm from the lens surface. *Scale bars*: 5 μm.

We next examined whether or not Cx46 distribution was affected in Cx50 KO lenses by imaging cross sections (Fig. 2B, Supplementary Fig. S2), anterior-posterior sections (Fig. 2C), and peeled fiber bundles (Fig. 2D). In WT samples, Cx46 was detected on the centers of the long and short sides of the fibers. Connexin 46 gap junctions appeared as large, uninterrupted plaques on BS and as smaller punctate spots on the flatter portions of the membranes. In Cx50 KO samples, we did not observe BS structures or Cx46 gap junction plaques with sizes similar to those of WT lenses (Figs. 2B–D, Supplementary Fig. S2B). Connexin 46 gap junctions appeared as numerous, smaller plaques, predominantly distributed around the centers of the long sides of the Cx50 KO fibers. Although typical BS structures were not detected in Cx50 KO lenses, we sometimes found WGA-positive vesicle-like structures abutting their fiber membranes. The structures are smaller than BS and appeared to be pinched at their base where they met the fiber membranes (Supplementary Fig. S3). These structures may represent small BS that do not mature normally without Cx50 and do not contain Cx46.

**Figure 3:**
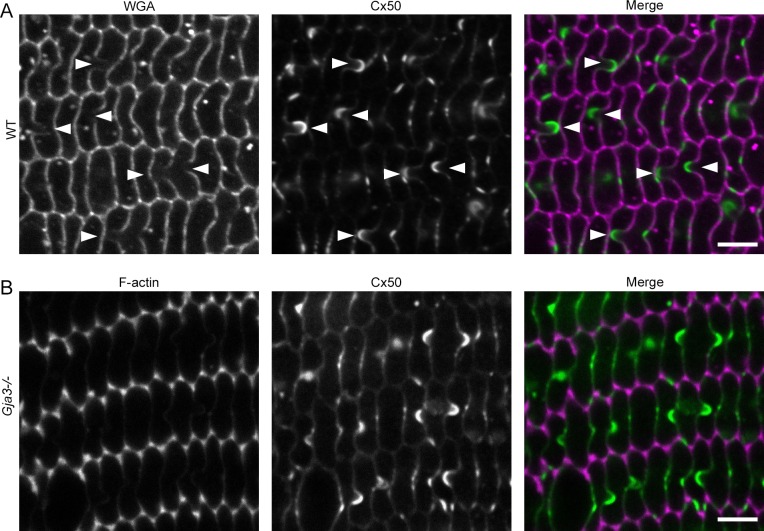
Ball-and-socket structures with Cx50 gap junctions are unchanged in Cx46 KO (*Gja3^−/−^*) fibers. (**A**) Connexin 50 is enriched on the BS structures of WT lens fibers. (**B**) Colabeling of F-actin and Cx50 shows presence of enriched F-actin in tricellular vertices and BS structures with Cx50 gap junctions in a Cx46 KO (*Gja3^−/−^*) section. *Scale bars*: 5 μm.

Similar to Cx46, Cx50 was also detected on the BS of WT samples (Fig. 3A). We further found typical BS with Cx50 gap junctions from 3-week-old Cx46 KO (*Gja3^−/−^*) lenses (Fig. 3B) and colabeling with phalloidin showed WT-like distribution of F-actin, including enrichment at tricellular vertices. Therefore, the disruption of F-actin and BS appears to be specific to loss of Cx50.

### Mislocalized ZO-1 in Cx50 KO Fibers

**Figure 4:**
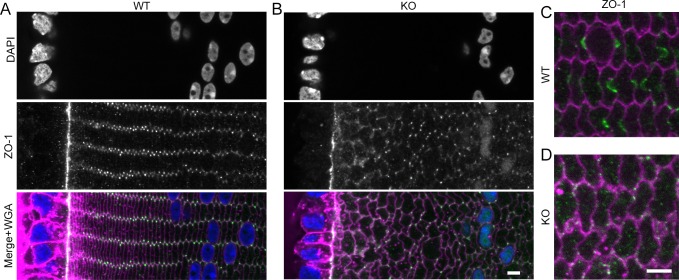
Distribution of ZO-1 in WT and Cx50 KO lenses. In the most peripheral fibers of WT (**A**) and KO (**B**) lenses, ZO-1 localizes to the epithelial-fiber interface and to tricellular vertices. However, in KO lenses, ZO-1 shows irregular distribution along fiber cell boundaries and in fiber nuclei. (**C**) Colabeling of ZO-1 (*green*) and WGA (*magenta*) shows that they are each enriched on BS in fibers near the denucleation zone. (**D**) Zonula occludens-1 is diffuse in Cx50 KO fibers at a similar depth. *Scale bars*: 5 μm.

We hypothesized that Cx50 gap junctions may interact with cytoskeletal proteins to maintain or stabilize BS structures. Zonula occludens-1 (ZO-1) was previously shown to interact with Cx46 and Cx50.^28,29^ Therefore, we determined the distribution of ZO-1 in lens fibers. Zonula occludens-1 staining in WT lenses was consistent with previous results: predominantly at the epithelial-fiber cell interface and on the vertices of the most peripheral fiber cells (Fig. 4A).^29^ Peripheral ZO-1 staining in Cx50 KO sections was similar, but was also diffusely over fiber cell boundaries and in fiber cell nuclei (Fig. 4B). Zonula occludens-1 began to appear on the WT BS approximately 150 to 175 μm in from the lens capsule (Fig. 4C), but ZO-1 was barely detected in the same region of Cx50 KO fibers (Fig. 4D). These data indicate that Cx50 is important for ZO-1 distribution in fiber cells. However, because ZO-1 appears on BS only in deeper fibers, it likely is not responsible for BS formation or stability.

### Beta-Dystroglycan and BS Structures

**Figure 5:**
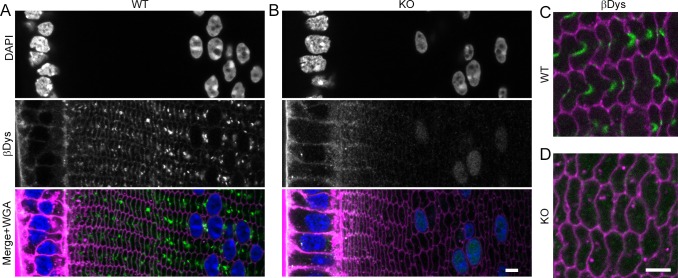
Distribution of βDys in WT and Cx50 KO lenses. (**A**) Beta-dystroglycan staining is strong on the membranes of epithelial cells, weak along fiber membranes, and strong on BS in WT lenses. (**B**) Beta-dystroglycan staining is similar in KO lenses except for the absence from BS and positive staining in fiber nuclei. Approximately 150 μm in from the lens capsule, βDys clearly labels BS in WT sections (**C**) but no specific localization is seen in KO sections (**D**). *Scale bars*: 5 μm.

We also hypothesized that certain integral membrane proteins could provide an asymmetric cue for establishing BS structures. We examined candidates that are expressed in lens fibers^30^ and are important for membrane structure in other cell types. We found that beta-dystroglycan (βDys) was predominantly localized to WT BS throughout peripheral fibers, similar to connexin staining, (Figs. 5A, 5C). In Cx50 KO lenses, βDys staining showed a diffuse signal on cell membranes as well as in the nuclei of peripheral fiber cells (Figs. 5B, 5D). Thus, βDys is one component of BS that relies on the presence of Cx50.

## Discussion

**Figure 6:**
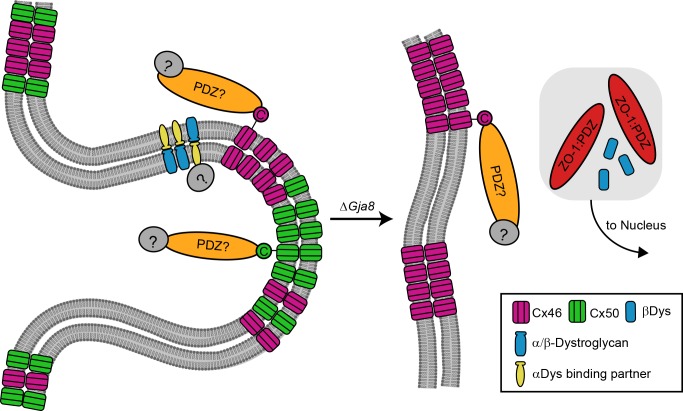
A conceptual model for the loss of BS structures between WT and Cx50 KO lenses. A BS is a specialized interlocking membrane domain that several proteins partition to in WT mice. Connexin C-terminals interact with unknown PDZ-domain–containing proteins (PDZ?) and their downstream binding partners. An extracellular binding partner for dystroglycan in the lens has yet to be identified. In Cx50 KO (Δ*Gja8*) lenses, BS are lost, Cx46-containing plaques are smaller in size, and at least a portion of the BS-associating proteins, ZO-1 and βDys, end up in fiber nuclei. Cytosolic, downstream partners for βDys and ZO-1 are also unknown. Ball-and-socket structure and gap junctions and proteins not drawn to scale.

Connexin 50 performs multiple tasks in the lens, including providing intercellular coupling, regulating epithelial cell proliferation, and promoting differentiation into fiber cells.^31–34^ In the present study, we showed disruption of BS with abnormal distribution of BS-associated proteins (Fig. 6) as well as irregular organization and altered F-actin distribution in Cx50 KO (*Gja8^−/−^*) fiber cells.

Ball-and-socket structures enriched with gap junctions are believed to provide rapid exchange of nutrients and metabolic signals during fiber cell elongation. The loss of Cx50 coupling with BS structures is likely to contribute to the smaller size of Cx50 KO lenses. Small, immature BS may still form in the Cx50 KO lenses, but do not contain Cx46. Biswas et al.^23^ observed small concavities with little to no connexins in embryonic chick lens fibers. Meanwhile, clathrin-coated concave membranes have been detected on nascent interlocking membrane domains, although further work is needed to determine whether clathrin is used for formation of protrusions, BS, or both.^35^ Therefore, initiation of BS may be independent of Cx50 gap junctions. Connexin 50 gap junctions may act during the maturation of BS by expanding and/or stabilizing surface curvature and BS size. The inherent structure of hemichannels can promote curvature by being narrower at one end of the channel than the other.^36^ Furthermore, it was previously suggested that, depending on the connexin packing symmetry and external membrane forces, curved junctions could be more stable than flat ones.^37^ It is also possible that Cx50 recruits other proteins to the membrane that induce curvature.^38,39^ The means by which connexins are trafficked and enriched to BS are unclear. Various connexins can preferentially associate with specific phospholipids, and these phospholipids can have increased inherent membrane curvature,^40^ but there is currently no evidence that the composition of lipids in BS is different from that in flat membrane regions. Also, curved membrane regions can passively promote the partitioning of specific proteins into them and reduce diffusion away from them, which could promote Cx50 enrichment.^41,42^

Scanning electron microscopy images of mouse lens fibers show variable BS densities,^22,43,44^ potentially due to differences in location of the fibers or the ages of the mice. It is unknown why and how BS become spatially restricted intracellularly and nonuniformly distributed throughout the lenses of many species despite Cx50 and Cx46 being broadly distributed on the short and broad sides of fibers. The distribution and size of gap junctions and mature BS may be associated with the posttranslational states of Cx50 and other BS-associated proteins. Lens connexins can undergo several posttranslational modifications^45,46^ that could influence the interactions of their C-terminal ends with cytosolic molecules such as PDZ-containing proteins (PSD95, Dlg1, and ZO-1) to promote partitioning to or away from BS (Fig. 6). It will be interesting to investigate what regulates the positioning, distribution, and size of BS in lens fibers at different ages and in different species.

The retention of BS and actin distribution in Cx46 KO (*Gja3^−/−^*) lenses highlights the nonredundant functions of Cx46 and Cx50. Ball-and-sockets and their associated factors seem less dependent on Cx46. The smaller Cx46 gap junction plaques in Cx50 KO lenses may be due to several factors, such as a loss of the aforementioned partitioning and retention of proteins at curved membranes due to loss of BS or a lower probability or propensity for accretion of Cx46 channels without Cx50. Equal quantities of Cx46 distributed in smaller plaques were previously shown to increase coupling resistance.^47^ Therefore, the observed changes in Cx46 gap junction assembly may contribute to a portion of the coupling deficits measured in Cx50 KO lenses.^48^

The disruption of F-actin with a reduction in enrichment at tricellular vertices of inner fibers provides a mechanistic clue about the disorganization and abnormal size of Cx50 KO fibers. Cytoskeletal integrity, especially the actin network, was previously shown to be critical to fiber cell migration, morphology, and packing.^49–55^ Disrupting the lens cytoskeleton also has effects on connexins. For example, lens-specific inhibition of Rho GTPases that regulate actin dynamics cause growth defects and concomitant decreases in Cx50 levels.^53,54^ Meanwhile, double KO of intermediate filament protein, CP49 (phakinin), and actin-capping protein, tropomodulin 1, increased coupling resistance while decreasing the size of Cx46-containing gap junctional plaques.^47^ This study demonstrates that the reverse case is also true: disrupting Cx50 leads to cytoskeletal changes. The mechanisms for reciprocal regulation between gap junctions and the cytoskeleton remain not well understood.^56,57^ In the case of Cx43, channel-dependent regulation and channel-independent regulation through interactions with actin-network–associated proteins are possible.^58–60^ Zonula occludens-1 is currently the only actin-associating protein known to interact with Cx50, but the actin-spectrin network is mostly excluded from beneath gap junctional plaques in cortical lens fibers.^47,61^ Further investigation with Cx50 mutants may provide more clues into the reciprocal regulation between Cx50 and the actin cytoskeleton.

Connexin 50Δ440 (a C-terminus deletion mutant) lenses display abnormal connexin distribution and identical phenotypes with Cx50 KO lenses.^28^ Unfortunately, Cx50Δ440 mutant mice were terminated (David Paul, Harvard Medical School, written communication, 2014). The C-terminus of Cx50 is essential for the function and assembly of Cx50 gap junctions. The multiple PDZ-domain–containing protein, ZO-1, can bind to the C-terminals of Cx50 and Cx46 in vitro.^29^ However, both our staining and previous results^29^ show extensive ZO-1 colocalization with gap junctions only in deep cortical fibers, after the assembly of BS structures with gap junction plaques, so the functional role of the interaction of ZO-1 with Cx50 or Cx46 on mature BS is unclear. Zonula occludens-1 may transiently associate with connexins for Cx50 or Cx46 trafficking in peripheral fibers. Connexin 50 was shown to prevent Skp2 nuclear localization by sequestering it in the cytosol.^21^ The detection of ZO-1 in Cx50 KO fiber nuclei points to the possibility that ZO-1 meant for interacting with Cx50 translocates into the nuclei. In some epithelial cells, ZO-1 nuclear accumulation is inversely related to the amount of cell-cell contacts.^62^ Currently, specific functions for ZO-1 in nuclei are unknown.^63^ Several other PDZ proteins, including Pdlim7, Htra3, Sipa1L3, and Afadin, are highly expressed in lens fibers based on RNA-seq data.^30^ However, we were unable to confirm their colocalization with gap junctions in fiber cells.

Beta-dystroglycan is a transmembrane protein that is typically linked with its extracellular binding partner, alpha-dystroglycan (αDys). Dystroglycan is widely expressed and provides both signaling and adhesive functions through interactions with a wide assortment of other proteins (e.g., actin, ezrin, and Src).^64,65^ It is perhaps most well known as a member of the dystrophin glycoprotein complex, where it provides mechanical integrity to cardiac and skeletal muscle.^66^ Little is known about βDys in the lens, but an important role is indicated by the fact that human dystroglycanopathies result in cataracts.^67^ Cell-cell adhesion is likely one role considering the connexin-like staining pattern we observed and its role in other cell types. In addition, βDys, αDys, and dystrophin are disrupted in ankyrin-B heterozygous mouse lens fibers, suggesting that βDys is regulated by the cytoskeleton.^68^ Like ZO-1, βDys contains a nuclear localization sequence and has been detected in the nuclei of several cell types,^65,69^ and there is some evidence that it can affect the nuclear matrix, nuclear envelope, and nuclear organization. Whether or not the actions of ZO-1 and βDys in the fiber nuclei contribute to the impaired nuclear degradation phenotype of Cx50 KO mice will require further study.

In summary, our results demonstrate that Cx50 is critical for BS structure, actin distribution, and fiber cell morphology. The disruption of BS in Cx50 KO (*Gja8^−/−^*) but not Cx46 KO (*Gja3^−/−^*) mice and decreased Cx46 plaque sizes provide new evidence that Cx50 gap junctional communication through BS is important for lens development, especially during rapid, early fiber cell growth. In addition to their roles in intercellular communication, BS gap junctions appear to function as specialized membrane domains for scaffolding of proteins, including ZO-1 and βDys, that could otherwise traffic to nuclei. Considering the myriad cytosolic partners of these proteins, it is likely that BS serve as a nexus for many signaling events, such as those associated with the cytoskeleton. Identification of additional BS-associating proteins is needed to understand what additional functions BS fulfill. Further studies are also needed to fully elucidate molecular bases for similarities and differences during BS and protrusion formation and maturation in lens fibers. Analysis of Cx50 KO, Cx50 point mutation, and knockin (Cx50KI46) lenses at different developmental time points and ages may provide additional mechanistic information about BS formation or maturation.

## Supplementary Material

Supplement 1
