# Supplementary material for: Connexin 50 Regulates Surface Ball-and-Socket Structures and Fiber Cell Organization
Source: Invest Ophthalmol Vis Sci. 2016 Jun 9;57(7):3039–46. doi: 10.1167/iovs.16-19521 (PMC4913802; doi:10.1167/iovs.16-19521)
Supplement: Supplement 1 [file i1552-5783-57-7-3039-s01.pdf]

Supplementary Material

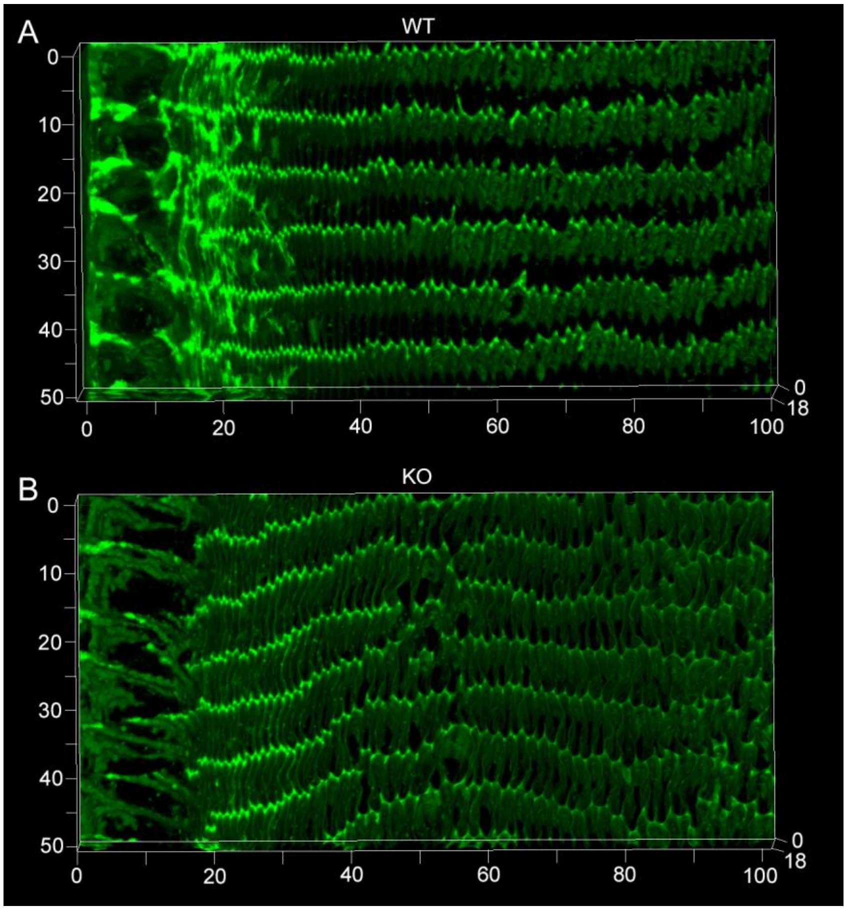

Supplementary Fig. 1. 3D rendering of F-actin labeled cortical fiber cross sections. (A) WT F-actin is concentrated at the hexagonal vertices of WT lens fibers and relatively weak on the long sides of the hexagons. (B) The F-actin is more evenly distributed in the fibers of Cx50 KO lenses, especially a noticeable reduction of enriched F-actin in tricellular vertices of inner fibers after ~50 μm in depth from the lens surface on the left side. 3D renderings are (100 μm x 50 μm x 18 μm).

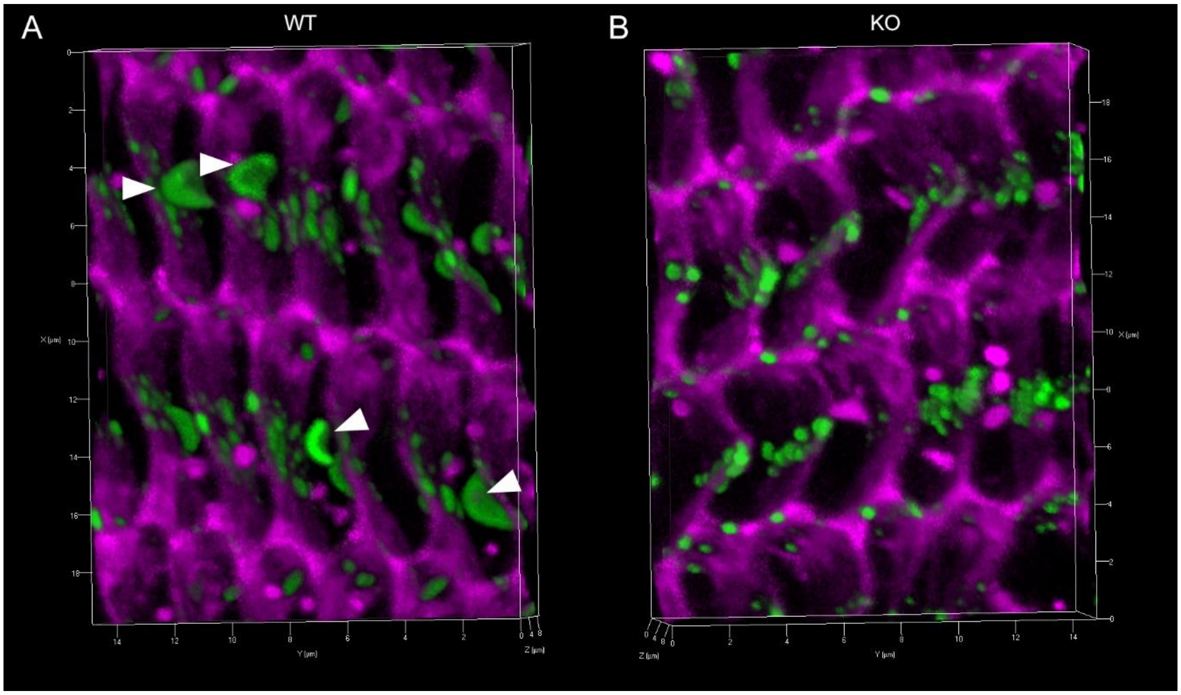

Supplementary Fig. 2. 3D rendering of WGA (magenta) and Cx46 gap junctions (green) labeled cross sections. (A) WT fibers have Cx46 labeling on BS (arrowheads) and punctate labeling on flatter membrane regions. (B) Cx50 KO fibers only have punctate Cx46 labeling. Images collected ~125 μm from the lens surface.

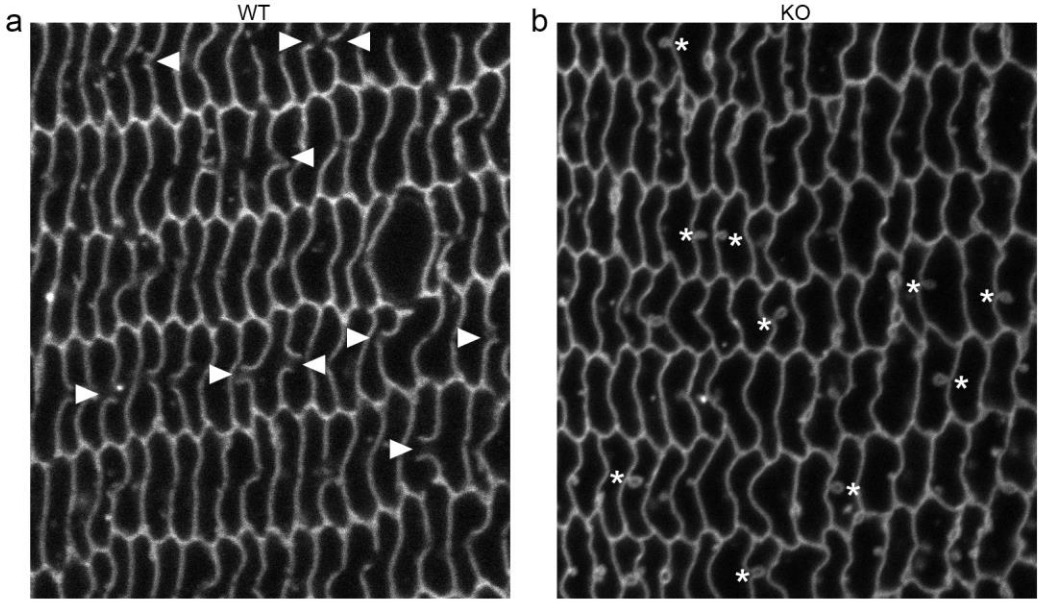

Supplementary Fig. 3. WGA labeling of WT and Cx50 KO cross sections. (A) BS on WT fibers have weak or absent WGA labeling except at their bases (arrowheads). (B) Some Cx50 KO fibers have small, WGA-positive vesicle-like structures abutting their membranes (asterisks). These structures may represent small immature BS that do not mature normally without Cx50 and do not contain Cx46. Images collected ~75 μm in from the lens surface.
